# Method for the mass production of seedlings of the tropical brown seaweed *Sargassum* (Phaeophyceae, Ochrophyta)

**DOI:** 10.1016/j.mex.2020.100854

**Published:** 2020-03-09

**Authors:** Danilo B. Largo, Gemlyn Mar S. Rance, Annie G. Diola, Jesrelljane Aaron-Amper

**Affiliations:** a Department of Biology, University of San Carlos, Talamban Campus, Talamban, Cebu 6000, Philippines; b Research, Development, Extension and Publications Office, University of San Carlos, Talamban Campus, Talamban, Cebu City, Philippines; c College of Fisheries and Marine Sciences, Bohol Island State University, Cogtong, Candijay, Bohol 6312, Philippines

**Keywords:** *Sargassum* hatchery, Flow-through system, Zygote recruitment, Artificial substrate, Clay or limestone substrates, Seeding string

## Abstract

Farming of *Sargassum* to produce harvestable crop can be a challenging task to seaweed farmers.

• Sexually-produced *Sargassum* seedlings can be propagated in a hatchery using 140-liter plastic tanks connected with PVC pipes and seawater supply directly pumped from the sea, passing through a filter system.
• First step of this method is to collect large amount of fertilized eggs from special branches called receptacles, found at the ends of lateral branches of *Sargassum*, excised from fertile thalli during its spawning season and collecting their eggs for recruitment into artificial substrate tanks.
• Egg collection involves force-releasing the fertilized eggs by vigorous shaking of a small vessel where 100-200 egg-bearing receptacles excised from fertile plants are contained. Each tank can produce up to 2000–3000 seedlings that can supply at least a hectare of farm. Scaling up the production to several hectares of farm is done by simply increasing the number of recruitment tanks and the number of recruitment panels in the hatchery system.

## Graphical abstract

**Figure:**
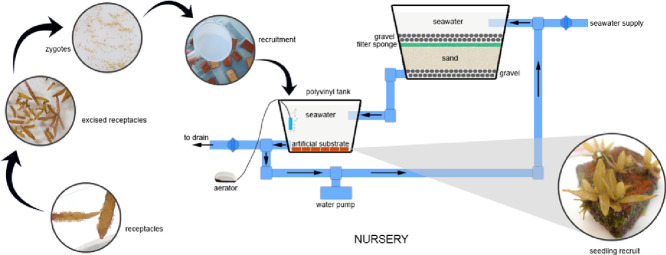

**Table:** Specification Table

Subject Area:	Agricultural and Biological Sciences
More specific subject area:	*Sargassum* seedling production
Method name:	Hatchery to produce seedlings of *Sargassum* from fertilized eggs
Name and reference of original method:	Invention disclosed to University of San Carlos and corresponding Patent filed at the Bureau of Patents, Intellectual Property of the Philippines bearing Invention Patent Application No. 12019050053 (Filing date: March 26, 2019)
Resource availability:	*N/A*

## Introduction

The brown seaweed *Sargassum,* which is abundant in the Philippines, is a source of livelihood for coastal communities. It is a source of chemical compounds with a wide range of applications in the pharmaceutical, biomedical, dental, textile, and printing industries among others. It is also a source of raw materials for extracting natural fertilizer and biostimulants [1]. *Sargassum* has also been found to have biosorptive capacity that has the potential to recover chemical pollutants and be reintegrated into the value chain – a prospect for the circular economy [2]. It used to be harvested from the wild or gathered from stranded materials at the beach after being washed ashore. Dense natural population of *Sargassum* serves as habitat, shelter, spawning and nursery grounds for myriads of marine organisms including important fishery resources [3]. Because of these ecosystem services, harvesting of *Sargassum* has been prohibited by Philippine law since 2014 (FAO 250 S. 2014) [4], affecting income of some coastal community who sell their harvested crop to traders, mainly for exportation. A small amount of the harvested *Sargassum* is processed locally to produce liquid fertilizer for agricultural applications [5]. As demand for *Sargassum* raw materials grow for both domestic and foreign markets, such as China, Japan and Korea, it is important that cultivation of this crop be developed to increase accessibility of the seaweed's available supply [6]. The method for cultivating *Sargassum* has been developed in China for sub-temperate and sub-tropical species and has been more or less adopted from the techniques used for the culture of kelps such as *Saccharina* and *Undaria* [7], [8], [9]. Basically this consists of seeding the fertilized eggs of *Sargassum* onto nylon string in an indoor tank under semi-controlled conditions [10], [11], [12], [13]. The attached zygotes are then allowed to develop into seedlings that, when big enough to withstand field cultivation, are attached to bigger culture ropes for out-planting in the field in floating long lines. Here we describe a slightly different technique for the mass-production of *Sargassum* seedlings based on different species of tropical *Sargassum*. This technique has been experimentally used to at least three different species of *Sargassum* for out-planting trials in different areas in southern Philippines.

## Method details

### Materials

• Utility boxes (140 L recruitment tank, 170 L filtration tank)
• PVC pipes (1.0-,3/4-, and 1/2-inch diameter)
• Valves
• Fine sand
• Gravel
• Aquarium-type filter -sponge
• Clay and limestone (Mactan stone) panels
• Nylon seeding string
• Plastic bottles, with cap, 100 ml
• Cutter
• Glass slides (roughened on one side)

### Equipment

• Air compressor
• Water pump
• Thermometer
• Refractometer
• Light meter
• Microscope
• Camera

### Preparation of the outdoor hatchery system

The land-based hatchery consists of a recirculating system set up in a semi-outdoor facility at the University of San Carlos Marine Research Station in Mactan Is., Cebu (Philippines). The hatchery system which at the same time serves as a nursery for the young recruits of *Sargassum* is composed of a filtration tank connected to a series of recruitment tanks made of plastic utility boxes (72 × 52 × 44 cm) supplied with filtered seawater that is sufficiently aerated and recirculated daily using a submersible water pump (see *Graphical Abstract*). The recruitment tanks which are simply made of plastic utility boxes can hold up to a maximum of 140 liters of seawater supplied from the adjacent sea. The seawater first has to pass through the filtration tank made of another plastic utility box (170 L volume) filled with a layer of gravel at the bottom, followed by fine sand at the middle, an aquarium filter sponge, and another gravel layer placed at the surface. The filter is intended to remove particulates and small algal contaminants (spores, gametes, fragments of adult thalli, etc.) from seawater. The recruitment tanks were semi-exposed to outdoor weather conditions, particularly to temperature and light. To avoid promoting the growth of certain algae, no addition of chemical fertilizer nor organic biostimulant is necessary.

### Preparation of recruitment substrates (panels and seeding string)

Substrate panels can be made of clay, limestone (a.k.a. Mactan stone) while seeding string can be of nylon material where zygotes are recruited. The clay panels can be made from broken clay pots, the limestone panels from Mactan stone tiles, and the nylon string (5 mm diameter) can be obtained from a fishing suuply store. The nylon string is prepared into a seeding substrate by wounding it around a rectangular PVC frame (48 × 32 cm). The density of recruited zygotes per square centimeter of panel can be determined based on their number divided by the area of each panel obtained by using *ImageJ* software (developed by the National Institutes of Health, U.S.A.), while the density of seedlings per string of the seeding string can be determined by counting the number of seedlings per area appearing on the entire seeding frame divided by the number of strings wound around the frame. The average densities of seedlings on panels and that on seeding string can then be compared with each other in terms of attachment efficiency. The clay and limestone panels are intended for bottom culture when *Sargassum* zygotes reach seedling size large enough to withstand open sea conditions. The seeding strings, on the other hand, are to collect recruits intended for floating culture. However, seedlings recruited on panels are found to be more stable than those on seeding strings which can be easily detached. Both panels and seeding strings are first immersed in seawater for roughly one week. This is to allow conditioning of the artificial substrates prior to their use, to allow natural bacterial film to coat all substrates that have the possibility to improve the recruitments of algae [14,15]. Each tank can accommodate at least 20 pieces of either clay or limestone panels to be placed at the bottom. Similarly, seeding strings wound in PVC frames can be placed at the bottom of each tank (*Graphical Abstract*). Glass slides (roughened on one side facing up) can be added together with the panels to facilitate observations under the microscope of the developing embryos up to the germling stage.

### Field collection of receptacles from fertile *Sargassum* plants and recruitment of zygotes unto artificial substrates

Prior to setting up the hatchery system, *Sargassum* beds from where receptacle branches are to be collected has to be checked first for the presence of fertile thalli which could vary from place to place between the months of October to January. Once fertile plants were ascertained to be egg-producing, the hatchery has to be immediately set into operation, or 24 h prior to field observations. This was to prevent any unforeseen kinks in the system and to avoid any delay as *Sargassum* spawns only within a window of 3–4 months in a year. In the field, receptacle branches are excised from fertile thalli, placed in Ziploc® bags, brought to the hatchery facility and placed into a white basin where they are first cleaned of epiphytes by brush and rinsed with filtered seawater. Receptacles are excised further from their main branches. Healthy and swollen receptacles with no obvious shedding are excised from the thalli and transferred to another basin containing sterile, filtered seawater. They are then grouped into 200 pcs and each group transferred into capped, transparent plastic bottle containing around 100 ml seawater which were then shaken vigorously to force-release the zygotes from the receptacles. The seawater containing the detached zygotes are then poured into another container through a fine mesh sieve, and the receptacles that remained in the sieve are returned into the container with seawater and shaken again a number of times to ensure maximum dislodgement of the zygotes. The zygote suspension can be immediately poured into the recruitment tank containing the substrate panels and seeding strings. To allow the zygotes to adhere firmly unto the substrates, there has to be no agitation of the seawater made for the first 48-72 h. Glass slides that are added together with the panels at the bottom can then be used to enable microscopic monitoring of the developing embryos that are not visible to the naked eye on the panels.

Observations could start as soon as the zygote suspension is poured into each tank, and their morphogenesis observed every 1–3 h under a light stereo-microscope (Motic SMZ-171). Glass slides are removed from the tank one at a time for the first 48 h. Images of developing embryos are captured using any camera such as from a smartphone or an underwater Olympus Tough TG-5 camera at an interval of about 3 h in the first 24 h, then daily until the embryos developed into germlings in the first week when they are visible enough to the naked eye on the different substrates. Monitoring is then continued every week in the succeeding weeks until germlings started to produce cauline leaves. By this time, they are called seedlings or plantlets. Culture condition for tropical *Sargassum* include water temperature ranging between 26–31 °C, salinity of 33–35, light intensity of not less than 50 µmol photons *m* ^−^ ^2^d^−1^. To monitor growth, photographs should be taken on representative seedlings with a ruler beside them as reference and the images saved directly into a computer from which size measurements can be conducted using free software like *ImageJ.* Photo-documentation need to continue at least every week until the seedlings increase in size that are ready for out-planting.

## Method validation

**Fig. 1:**
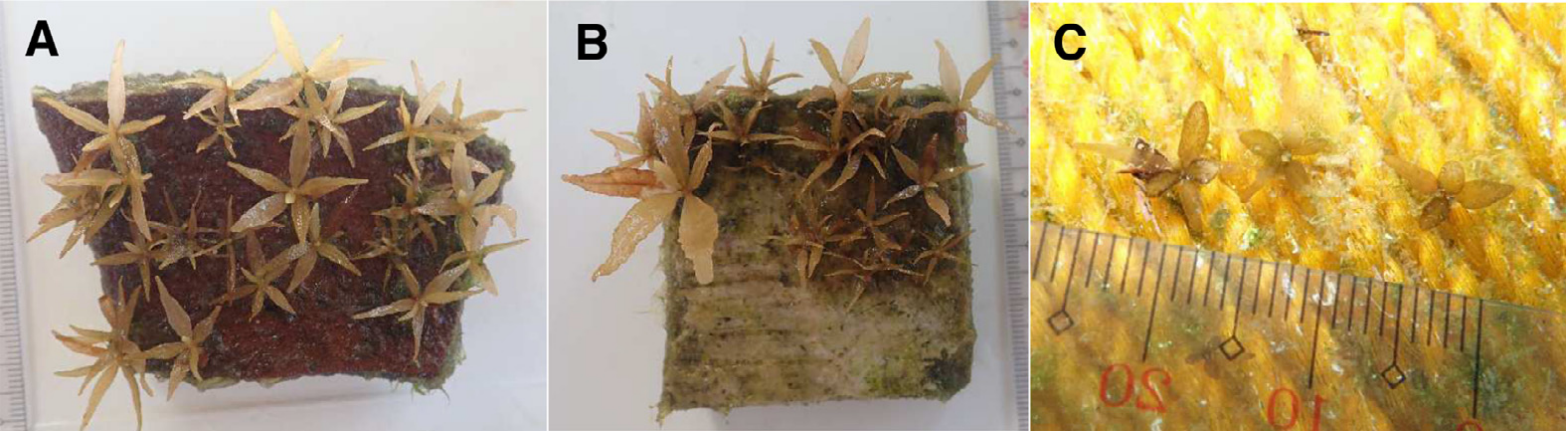
Substrates bearing *Sargassum* recruits/seedlings: (A) – clay panel, (B) – limestone panel, (C) – nylon string.

Hatchery system using plastic utility boxes, PVC pipes and valves and with air compressors and water pump, can be inexpensive materials to mass-produce *Sargassum* seedlings of a few thousands per box without addition of fertilizer or biostimulant. Density of seedlings from 200 receptacles recruited onto artificial substrate made of clay and limestone panels was found to produce high seedling densities (Fig. 1). This can be adjusted by the number of receptacles used to obtain zygotes for recruitment per tank.

**Fig. 2:**
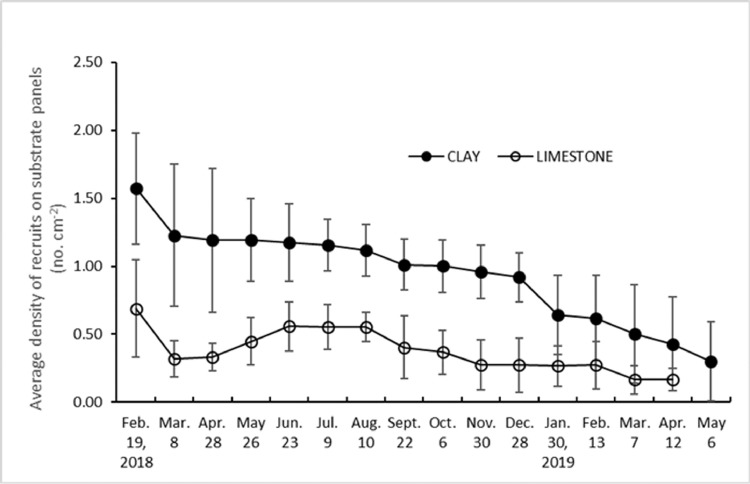
Recruitment of *Sargassum* zygotes onto artificial substrates made of clay and limestone panels showed an initially high average density of the recruits but gradually decreased if incubation in tanks is prolonged.

Note: We transferred the seedlings for out-planting to the sea after more than a year in the tanks when no further growth of the seedlings was observed.

Between the two substrate panels used, clay panels proved to be more efficient for recruitment of *Sargassum* seedlings where average density can be as high as 1.57 recruits cm^−2^ compared to that of limestone which obtained only an average of 0.69 recruit cm^−2^. This, however could decrease to just 0.30 and 0.17 recruit cm^−2^ respectively, after a year (Fig. 2). Meanwhile recruits on seeding string made of nylon lacked the ability to hold seedlings for a long incubation period (result not anymore shown).

## Conclusion

The hatchery system – consisting of recruitment tanks supplied with filtered seawater that is recirculated – enables the production of *Sargassum* seedlings for out-planting. Vigorous agitation of 100–200 receptacles in a container force-releases a substantial quantity of fertilized eggs that are able to recruit on artificial substrates placed at the bottom of the tanks. Among the three types of materials used as artificial substrates, clay panels proved to be more efficient than limestone panel and nylon seeding string based on density of recruits per cm^2^ and stability of adhesion for more than a year maintained within optimal conditions of temperature, salinity and light intensity. This hatchery system can be scaled up to produce a desired number of seedlings by adding more recruitment tanks and substrate panels.
